# Endogenous reactive oxygen species and nitric oxide have opposite roles in regulating HIF-1alpha expression in hypoxic astrocytes

**DOI:** 10.52601/bpr.2021.200016

**Published:** 2021-06-30

**Authors:** Qingquan Chen, Wenlan Liu, Xi Sun, Ke Jian Liu, Rong Pan

**Affiliations:** 1 Department of Pharmaceutical Sciences, University of New Mexico Health Sciences Center, Albuquerque, NM 87131, USA

**Keywords:** Hypoxia, Hypoxia-inducible factor 1, Astrocyte, Nitric oxide, Reactive oxygen species

## Abstract

Ischemic stroke results in cerebral tissue hypoxia and increased expression of hypoxia-inducible factor (HIF), which is critically implicated in ischemic brain injury. Understanding the mechanisms of HIF-1alpha regulation in the ischemic brain has been an important research focus. The generation of both nitric oxide (NO) and reactive oxygen species (ROS) is increased under hypoxic/ischemic conditions and each of them has been independently shown to regulate HIF-1alpha expression. In this study, we investigated the cross-effects of NO and ROS on the expression of HIF-1alpha in hypoxic astrocytes. Exposure of astrocytes to 2 h-hypoxia remarkably increased HIF-1alpha protein levels, which was accompanied by increased NO and ROS production. Decreasing ROS with NAC, NADPH oxidase inhibitor DPI, or SOD mimetic MnTMPyP decreased hypoxia-induced HIF-1alpha protein accumulation and increased NO level in hypoxic astrocytes. The NO synthase (NOS) inhibitor L-NAME inhibited ROS generation, which led to a reduction in hypoxia-induced HIF-1alpha protein expression. Although NOS inhibitor or ROS scavengers alone reduced HIF-1alpha protein levels, the reduction was reversed when NOS inhibitor and ROS scavenger present together. The NO scavenger PTIO increased hypoxia-induced HIF-1alpha protein expression and ROS production, while HIF-1alpha protein level was decreased in the presence of NO scavenger and ROS scavenger together. These results suggest that ROS, NO, and their interaction critically contribute to the regulation of hypoxia-induced HIF-1alpha protein accumulation under hypoxic condition. Furthermore, our results indicate that hypoxia-induced NO generation may represent an endogenous mechanism for balancing ROS-mediated hypoxic stress, as reflected by inhibiting hypoxia-induced HIF-1alpha protein accumulation.

## INTRODUCTION

Hypoxia is a cardinal characteristic of ischemic stroke, and the expression of the hypoxia-inducible transcription factor 1 (HIF-1) plays a critical role in the cellular response to hypoxic conditions through regulating the transcription of more than 100 genes (Jung *et al*. 2004). HIF-1 is a heterodimer, which is composed of subunits of HIF-1alpha and HIF-1beta (Wang and Semenza 1995). While the beta subunit is continuously expressed, the alpha subunit is rapidly degraded in the presence of oxygen under normoxic conditions. This occurs through a degradation reaction controlled by prolyl hydroxylase domain-containing enzymes (PHDs) (Bruick and McKnight 2001; Maxwell and Eckardt 2016; Oehme *et al*. 2002). Accumulating evidence indicates that increased ROS generation critically contributes to hypoxia-induced HIF-1alpha protein elevation (Galanis *et al*. 2008; Guzy *et al*. 2005; Wang *et al*. 2018). Genetic or pharmacological interventions that enhance or reduce ROS, have been shown to enhance or reduce the accumulation of HIF-1alpha protein, respectively (Brunelle *et al*. 2005; Guzy *et al*. 2005; Wang *et al*. 2018). ROS have been shown to stabilize HIF-1alpha protein via direct inhibition of the PHD enzyme, by reducing the availability of the co-factors of this enzyme (including ascorbate, Fe(II), Krebs cycle intermediates, or through disulfide bond-mediated prolyl hydroxylase domain protein 2 dimerization), or by regulating SHP-1 (Alig *et al*. 2015; Chandel and Budinger 2007; Lee *et al*. 2016; Moudgil *et al*. 2005; Pouyssegur and Mechta-Grigoriou 2006).

In addition to ROS, nitric oxide (NO) is also a key player in regulating HIF-1alpha expression. Studies indicated that under normoxic conditions, increased NO production from NO donors or inducible NO-synthase enhance HIF-1alpha stability as well as increased its mRNA transcription (Ball *et al*. 2012; Kasuno *et al*. 2004; Kurokawa *et al*. 2019; Sandau *et al*. 2001; Yamamoto *et al*. 2020). NO has also been shown to increase HIF-1alpha protein via inhibiting PHD enzyme (Metzen *et al*. 2003). However, under hypoxic condition, NO appears to inhibit HIF-1alpha expression (Berchner-Pfannschmidt *et al*. 2007; Kozhukhar *et al*. 2006). NO was shown to inhibit cytochrome c oxidase and thus inhibits mitochondrial oxygen consumption, which allowed enough oxygen available for PHD enzymes to regain its activity to degrade HIF-1alpha protein (Hagen *et al*. 2003). Additionally, iron and calcium were also believed to be involved in the inhibition effect of NO on HIF-1alpha under hypoxic conditions (Callapina *et al*. 2005; Zhou *et al*. 2006). Unfortunately, the observed inhibitory effect of NO on hypoxia-induced HIF-1alpha protein elevation was derived mainly from studies on exogenous NO; it remains unclear whether increased endogenous NO production affects HIF-1alpha expression under hypoxic conditions.

Both NO and ROS are important factors involved in the cellular response to hypoxia (Rodrigo *et al*. 2004; Pan *et al*. 2019; Semenza and Prabhakar 2018; Siques *et al*. 2018). In addition, it is also known that NO can rapidly react with superoxide to form a more potent oxidant peroxinitrite (Ridnour *et al*. 2008). In the present study, we investigated the opposing effects of endogenous NO and ROS, and their interaction, on hypoxia-induced HIF-1alpha protein stabilization. Moreover, accumulating evidence indicates that NADPH oxidase is an important source of ROS under hypoxic or ischemic conditions (Kahles *et al*. 2007; Liu *et al*. 2008; Moon *et al*. 2010; Pan *et al*. 2019; Zhao *et al*. 2018). Therefore, in this study, we also tested if NADPH oxidase played a role in the stabilization of HIF-1alpha.

## RESULTS

### Effects of endogenous ROS on the expression of HIF-1alpha

**Figure 1:**
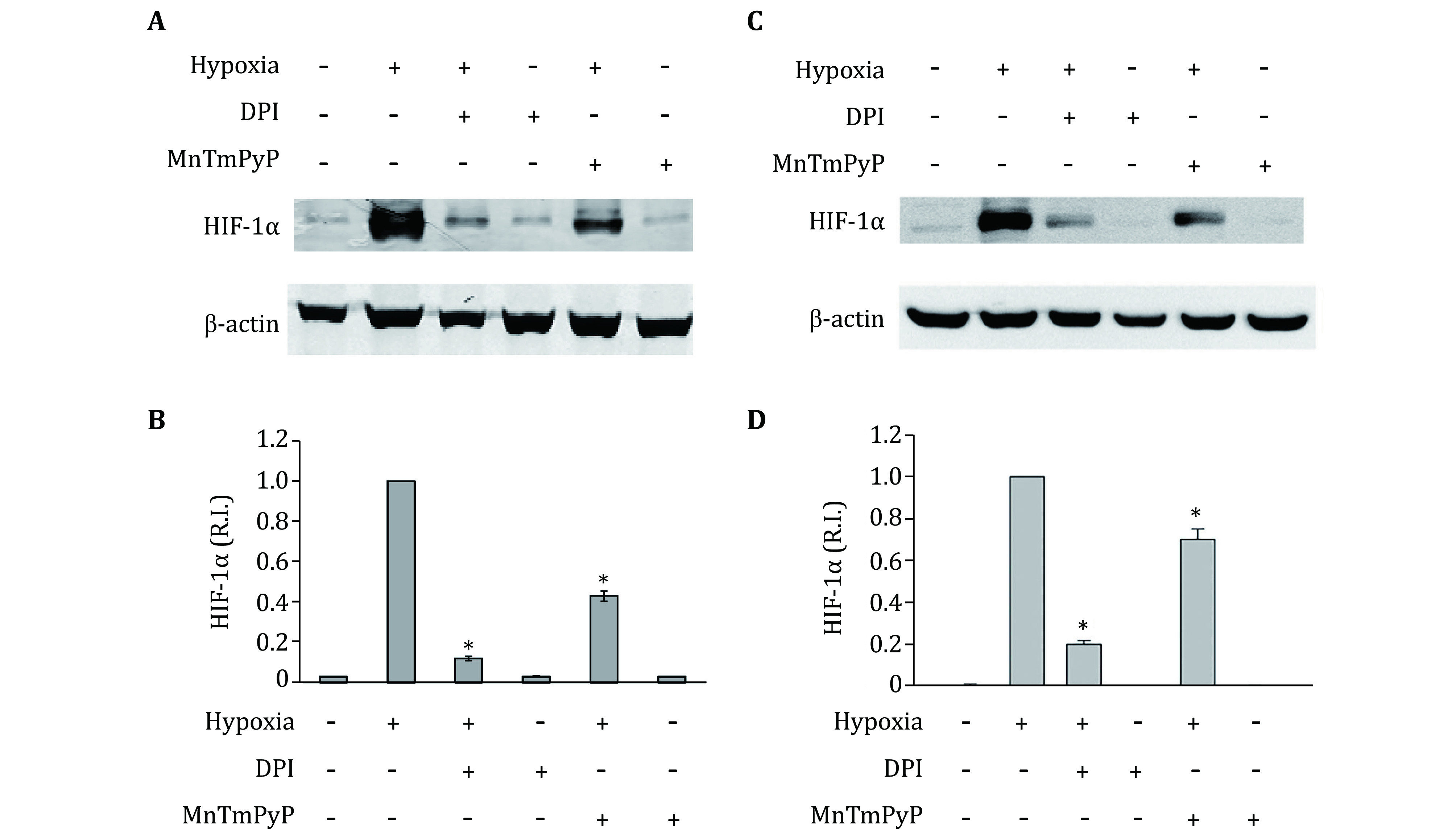
Effects of DPI and MnTMPyP on the expression of HIF-1alpha in primary astrocytes (**A, B**) and C8-D1A cells (**C, D**). **A, C** A representative Western blot. **B, D** Quantitative data of HIF-1alpha protein levels. Primary astrocytes or C8-D1A cells were exposed to normoxia or hypoxia for two hours after 20 min pretreatment with 30 μmol/L DPI or 5 μmol/L MnTMPyP. The protein band intensity was quantitated and normalized to the hypoxia group (R.I.: relative intensity). Data were expressed as mean ± SEM (*n* = 3). *p < 0.05 compared with the hypoxia group

Our recent studies show a significant increase of endogenous ROS in hypoxic astrocytes (Pan *et al*. 2019). To demonstrate that endogenous ROS plays an important role in hypoxia-induced HIF-1alpha expression, the NADPH oxidase inhibitor DPI and cell permeable SOD mimetic MnTMPyP were used to decrease intracellular ROS production. As shown in Fig. 1A and 1B, the addition of DPI or MnTMPyP did not change HIF-1alpha protein levels in normoxic primary astrocytes. Hypoxia treatment for 2 h significantly increased HIF-1alpha protein levels in the primary astrocytes. Notably, unlike under normoxic condition, the addition of DPI or MnTMPyP markedly inhibited the hypoxic-induced HIF-1alpha protein elevation. We obtained similar results in astrocytic cell line C8-D1A (Fig. 1C and 1D). These results indicate that endogenous ROS production contributes to hypoxia-induced HIF-1alpha protein elevation in astrocytes.

### Effects of NOS or NO inhibition on the expression of HIF-1alpha

**Figure 2:**
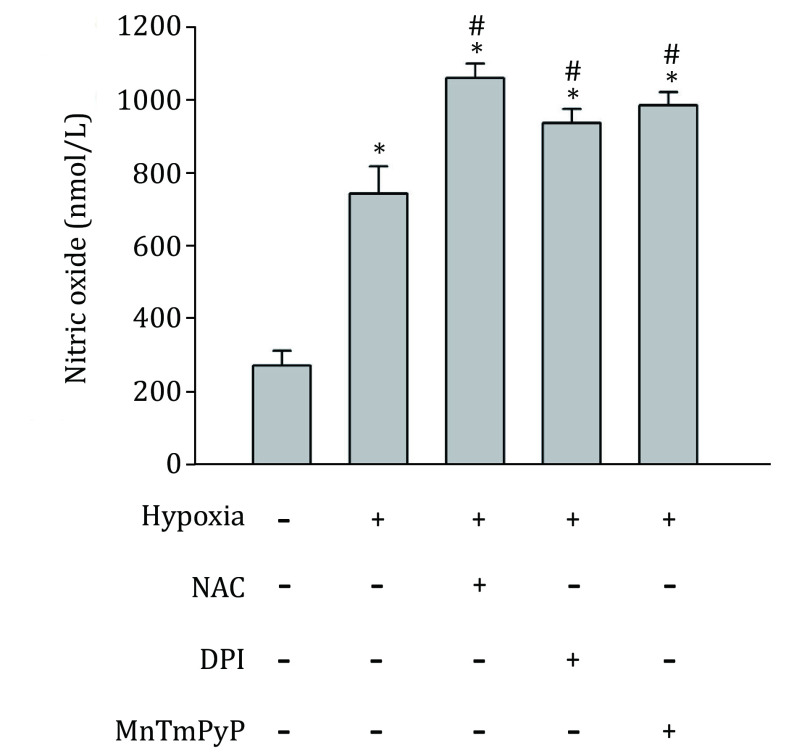
Effects of DPI, MnTMPyP and NAC on hypoxia-induced NO production. C8-D1A cells were exposed to normoxia or hypoxia for two hours after 20 min pretreatment with or without the indicated ROS inhibitor or scavenger. Hypoxia increased NO generation, which was further increased in the presence of NAC (1 mmol/L), DPI (30 μmol/L) or MnTMPyP (5 μmol/L). Data were expressed as mean ± SEM (*n* = 3). *p < 0.05 compared with normoxia group; #*p* < 0.05 compared with hypoxia group

**Figure 3:**
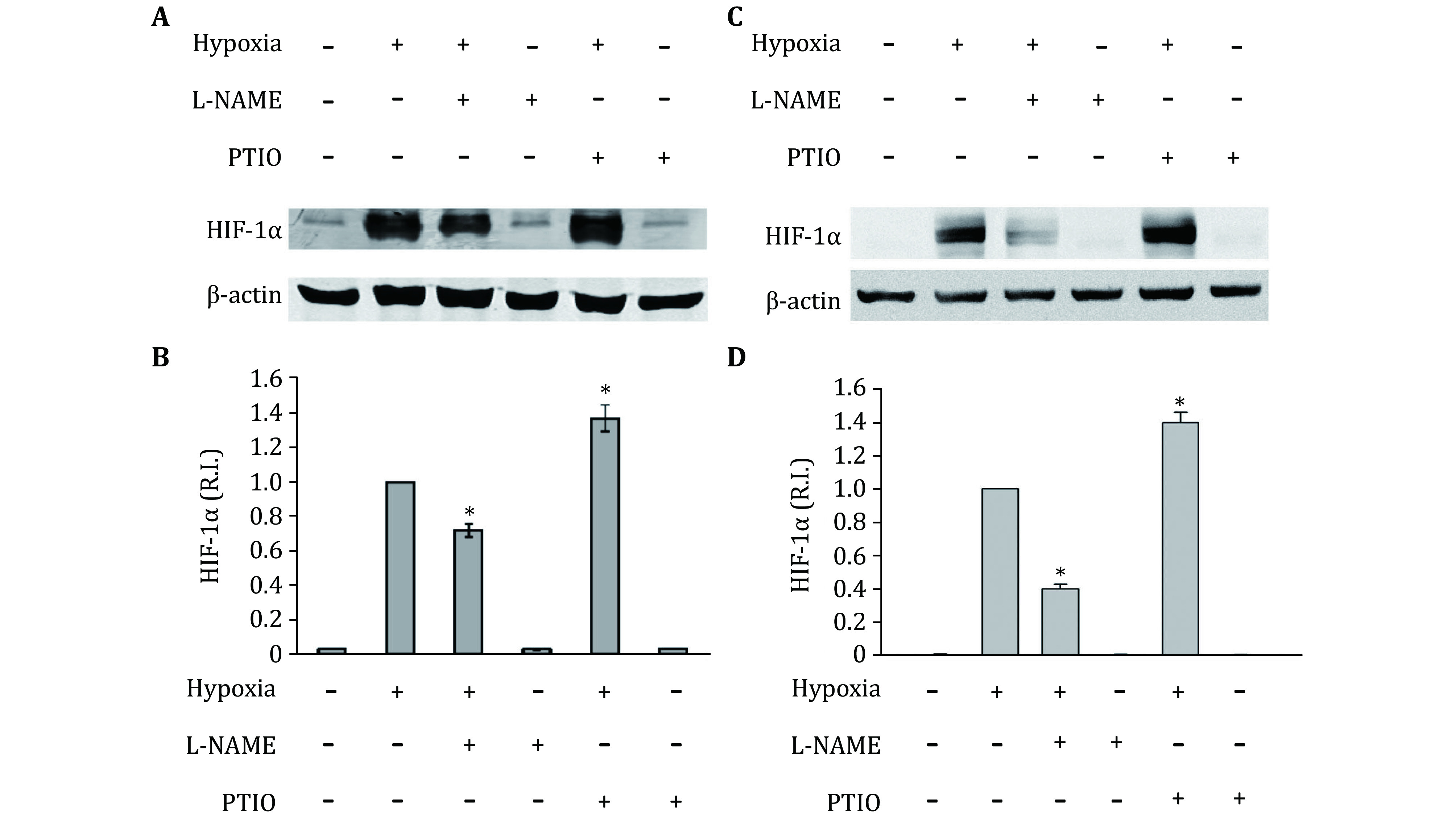
Effects of L-NAME and PTIO on the expression of HIF-1alpha in primary astrocytes (**A, B**) and C8-D1A cells (**C, D**). **A, C** A representative Western blot. **B, D** Quantitative data of HIF-1alpha protein levels. Primary astrocytes or C8-D1A cells were exposed to normoxia or hypoxia for two hours after 20 min pretreatment with 1 mmol/L L-NAME or 100 μmol/L PTIO. The protein band intensity was quantitated and normalized to the hypoxia group (R.I.: relative intensity). Data were expressed as mean ± SEM (*n* = 3). *p < 0.05 compared with the hypoxia group

Under hypoxic conditions, inducible NOS has been reported to be upregulated in astrocytes (Guo and Bhat 2006), indicating an increase of NO in hypoxic astrocytes. To confirm the generation of endogeans NO, inNO-T nitric oxide measurement system was used to measure NO concentration. As show in Fig. 2, NO level significantly increased in hypoxic astrocytes. Then, to determine the effects of endogenous NO on the expression of HIF-1alpha under hypoxic conditions, primary astrocytes were treated with NOS inhibitor L-NAME or NO scavenger PTIO (Okamoto *et al*. 2002; Thomsen *et al*. 1994). Neither PTIO nor L-NAME changed HIF-1 protein level under normoxic condition. Interestingly, under hypoxic condition, treatment with PTIO significantly increased hypoxia-induced HIF-1alpha protein level, while, L-NAME inhibited HIF-1alpha protein elevation in both primary astrocytes and astrocytic cell line C8-D1A (Fig. 3). Since the results from primary astrocytes and cell line C8-D1A are almost identical, we used C8-D1A cells to perform all subsequent experiments.

### Effects of NOS or NO inhibition on ROS production

**Figure 4:**
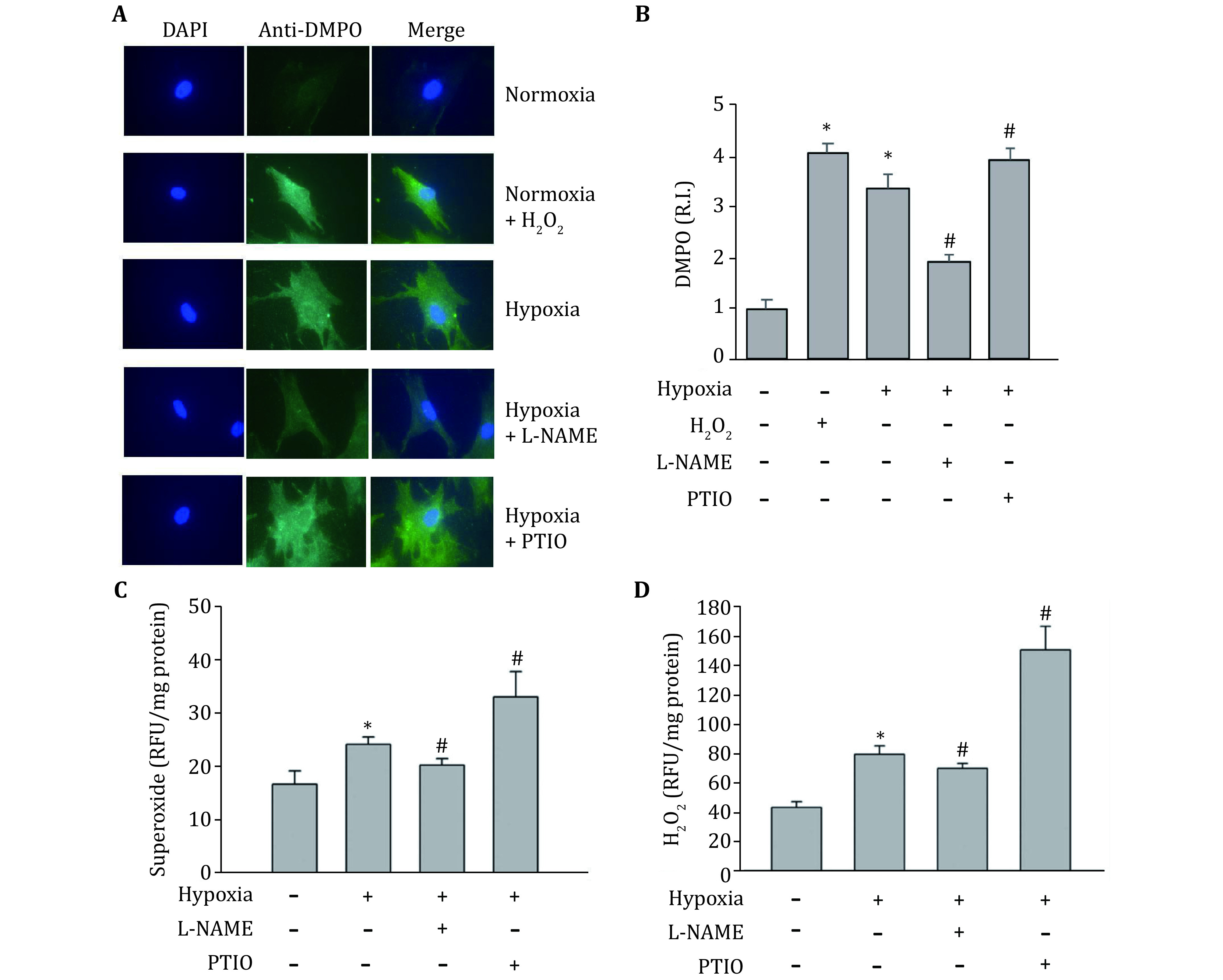
Effects of L-NAME and PTIO on hypoxia induced ROS production. At the end of the indicated treatments, protein-derived superoxide was measured by immuno-spin trapping (**A**). H_2_O_2_ was used as positive control in immune-spin trapping. The relative intensity (R.I.) of DMPO signal was quantitated and normalized to the normoxia group (**B**). The intracellular superoxide was measured by DHE staining (**C**), while hydrogen peroxide levels were measured by DCFH-DA staining (**D**) as described in the method section. Values of the relative fluorescence unit (RFU) were normalized to the protein concentrations. Data were expressed as mean ± SEM (*n* = 3). *p < 0.05 compared with the normoxia group; #*p* < 0.05 compared with hypoxia group

Because NOS is not only a source of NO, but also a source of ROS, especially under pro-oxidant (Huetsch *et al*. 2019; Landmesser *et al*. 2003) and hypoxia (Muzaffar *et al*. 2005) conditions, we speculated that the observed opposite effects of NOS inhibitor (L-NAME) and NO scavenger (PTIO) on HIF-1alpha expression might result from their different impacts on ROS production. In order to confirm this speculation, we measured their effects on superoxide and hydrogen peroxide production under hypoxic conditions. DMPO, a spin trap for superoxide, was used to detect protein-derived superoxide utilizing the immuno-spin trapping method. As shown in Fig. 4A and 4B, the signal of protein-derived superoxide increased after hypoxia. PTIO amplified the increase of protein-derived superoxide, while L-NAME decreased protein-derived superoxide level. To confirm this result, DHE staining was used to measure the intracellular superoxide. As shown in Fig. 4C, just like the results from immune spin-trapping, the production of superoxide significantly increased in hypoxic astrocytes, which further increased by PTIO treatment, while decreased by L-NAME treatment. The hydrogen peroxide staining with DFH-DA was used to measure the intracellular hydrogen peroxide. Similar to superoxide, exposure of astrocytes to hypoxia for 2 h remarkably increased the production of hydrogen peroxide, which was further augmented in the presence of PTIO while attenuated in the presence of L-NAME (Fig. 4D). PTIO increase ROS production presumably through removing NO, thus prevent NO from reacting with superoxide. On the other hand, although treatment with L-NAME should reduce NO production, it also could inhibit NOS-derived ROS, leading to an overall observed reduction in the levels of superoxide and hydrogen peroxide.

### Effects of endogenous NO on the expression of HIF-1alpha

**Figure 5:**
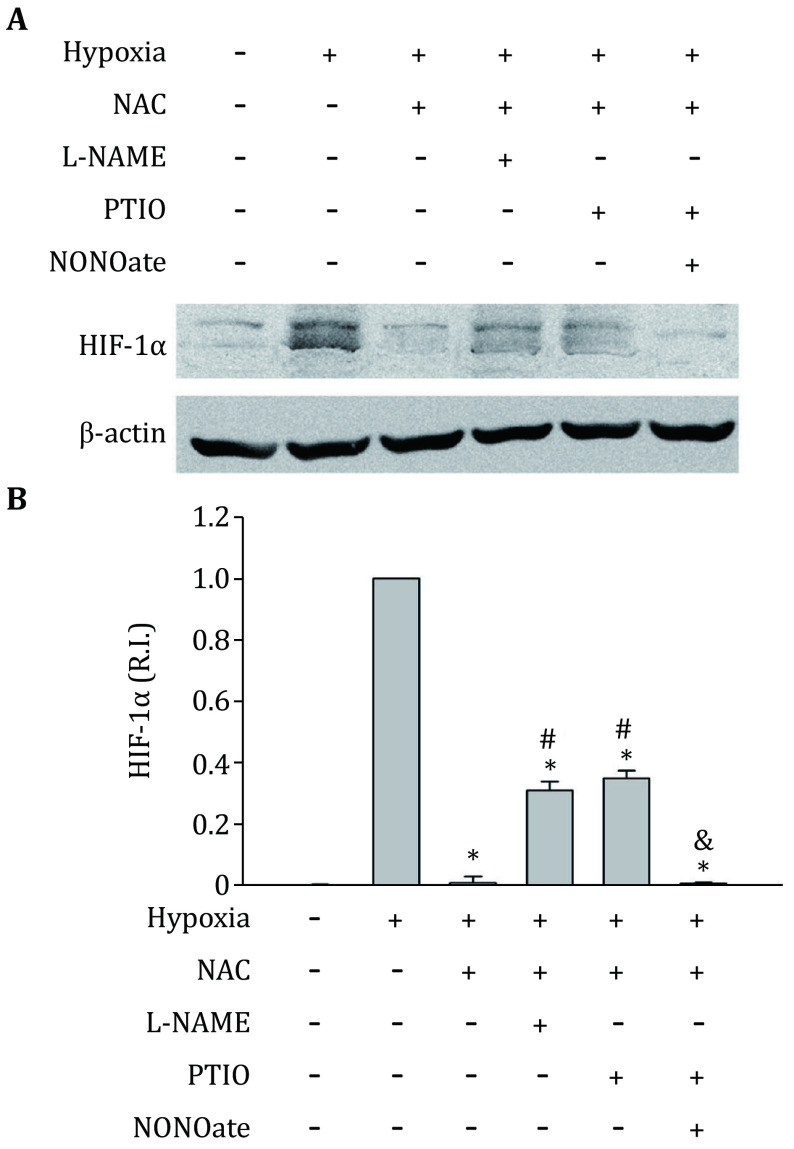
Effects of L-NAME and PTIO on HIF-1alpha expression in hypoxic C8-D1A cells pretreated with NAC. **A** A representative Western-blot. **B** Quantitative data of HIF-1alpha protein levels. 1 mmol/L NAC almost completely abolished the HIF-1alpha protein accumulation induced by hypoxia treatment. Both L-NAME (1 mmol/L) and PTIO (100 μmol/L) increased HIF-1alpha protein levels compared to the hypoxia + NAC group. The protein band intensity was quantitated and normalized to the hypoxia group (R.I.: relative intensity). Results were expressed as mean ± SEM (*n* = 3). *p < 0.05 compared with hypoxia group; #*p* < 0.05 compared with hypoxia + NAC group; &*p* < 0.05 compared with hypoxia+NAC+PTIO group

Because of the interaction of ROS with NO, to elucidate the direct effect of NO on HIF-1alpha expression, we have to remove the effect of ROS. Thus, a ROS scavenger (NAC 1 mmol/L) was used to remove ROS in this study. Treatment with NAC almost completely abolished HIF-1alpha protein elevation induced by hypoxia (Fig. 5). However, the combined treatments of NAC + L-NAME, or NAC + PTIO, resulted in higher HIF-1alpha protein levels than NAC alone (Fig. 5). These results suggest that while endogenous ROS generation increases hypoxia-induced HIF-1alpha protein level, the NO production may have an opposite and direct inhibitory effect on HIF-1alpha protein, which is demonstrated by the findings that in the presence of ROS scavengers NAC, removing NO stabilizes hypoxia-induced HIF-1alpha protein. To further confirm this conclusion, NAC and PTIO were used to treat C8-D1A cells to make an endogenous ROS and NO free conditions. Then, NO donor NONOate was added to C8-D1A cells to induce exogenous NO and exposed to hypoxia for 2 h. Under these conditions, HIF-1alpha protein decreased to its low basal level by exogenous NO addition (Fig. 5). The results indicate that NO decreases HIF-1alpha protein expression.

### Effect of ROS on the production of NO

Besides the regulation of ROS level by NO production, ROS generation also regulates NO level (Hsieh *et al*. 2014). Thus, we investigated the effect of ROS on NO production in hypoxic C8-D1A cells in the presence or absence of ROS inhibitor or scavengers. Two hours of hypoxic treatment increased NO concentration, which was further increased by NADPH oxidase inhibitor DPI, SOD mimetic MnTMPyP, or the antioxidant NAC (Fig. 2). These results indicate that, under hypoxic condition, reducing ROS production may increase the bioavailability of NO.

### Effects of peroxynitrite on the HIF-1alpha expression under hypoxic conditions

**Figure 6:**
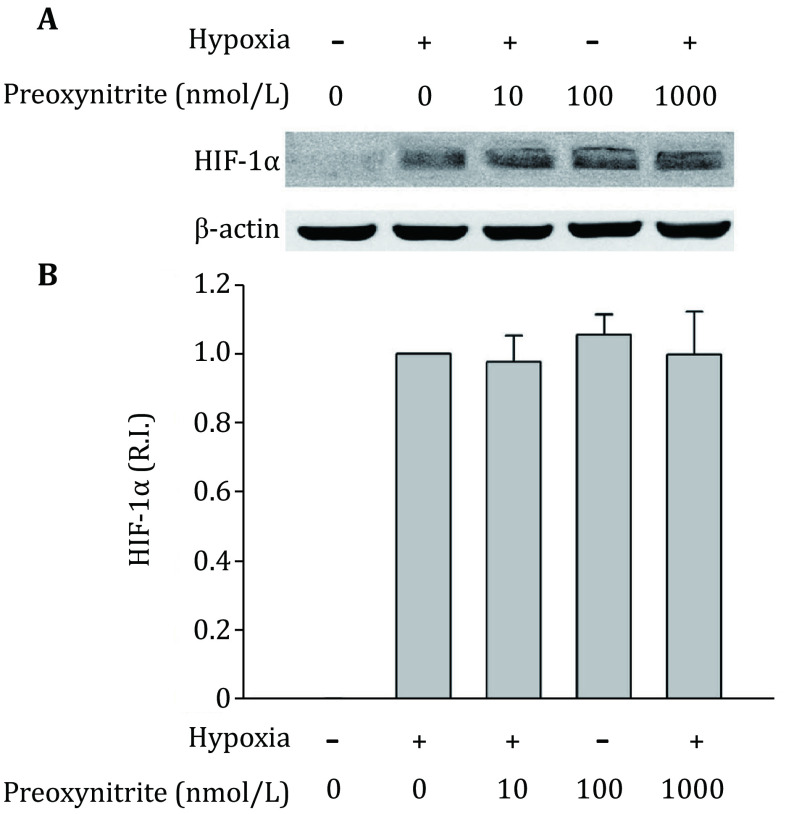
Effects of peroxynitrite on the expression of HIF-1alpha. **A** A representative western-blot. **B** Quantitative data of HIF-1alpha protein levels. C8-D1A cells were treated with different concentrations of peroxynitrite under hypoxic conditions. The protein band intensity was quantitated and normalized to the hypoxia group (R.I.: relative intensity). Results were expressed as mean ± SEM, *n* = 3

The above experiments showed that, under hypoxic condition, NO decreased HIF-1alpha expression, while ROS increased it. Since NO can rapidly react with superoxide to produce peroxynitrite, it would be important to determine whether peroxynitrite affects HIF-1alpha expression. Right before hypoxia treatment, we added different concentrations of peroxynitrite donor SIN-1to cells. As shown in Fig. 6, incubation C8-D1A cells with peroxynitrite did not significantly change HIF-1alpha protein levels. It indicates that although the cross regulation of NO and ROS significantly affect HIF-1alpha protein levels (Fig. 2, 4 and 5), the potential product of NO and superoxide, peroxynitrite, does not affect HIF-1alpha protein levels.

NO has been reported to be an antioxidant. The near diffusion control of ROS scavenging by NO may be a direct and significant mechanism for the antioxidant property of NO (Arora *et al*. 2016; Ridnour *et al*. 2008). Our results that elimination of NO with PTIO greatly increased ROS (Fig. 4), as well as hypoxia-induced HIF-1alpha protein accumulation (Fig. 3 and 5), suggest that the NO-ROS interaction contributes to the regulation of HIF-1alpha expression under hypoxic condition. This is consistent with the reports that scavenging of ROS with exogenous NO reduced HIF-1alpha expression (Agani *et al*. 2002; Kohl *et al*. 2006). To determine the direct effects of endogenous NO on HIF-1alpha regulation, we excluded the influence of ROS through the use of a ROS scavenger NAC. We found that under the combined hypoxia + NAC conditions, eliminating NO by either L-NAME or PTIO increased HIF-1alpha expression. Moreover, the addition of NO to the system abolished the effects of PTIO (Fig. 5). These results indicate that the endogenous NO directly inhibits hypoxia-induced HIF-1alpha protein accumulation.

## DISCUSSION

Hypoxia plays an important role in the pathophysiology of ischemia-induced brain injury. Although HIF-1 has been a research focus for many years, the regulation of HIF-1 is still not fully understood. Our present study demonstrates that endogenous ROS increases, whereas endogenous NO decreases, HIF-1alpha level under hypoxic condition.

HIF-1 is a heterodimer, consisting of constitutively stable HIF-1beta and oxygen-sensitive HIF-1alpha subunits (Wang and Semenza 1995). Both HIF-1alpha and HIF-1beta are continuously transcribed and translated, but under normoxic conditions, the HIF-1alpha subunit is hydroxylated at conserved proline residues in an oxygen-dependent domain by PHDs, followed by ubiquitylation and rapid degradation by the ubiquitin-proteasome system (Bruick and McKnight 2001; Oehme *et al*. 2002). Thus, the regulation of HIF-1 activity takes place through changes to the alpha subunit. In addition to oxygen, other factors like ROS and NO are implicated in the regulation and degradation of HIF-1alpha.

The role of ROS in HIF-1alpha regulation under hypoxic conditions has been extensively studied, and a mitochondrial response theory has been proposed, in which mitochondria responds to the hypoxic stimulus to produce a burst of ROS that are both necessary and sufficient to stabilize HIF-1alpha (Chandel *et al*. 1998). However, a later study showed that cells lacking mitochondrial function (p^0^ cells) were still capable of stabilizing HIF-1alpha in response to hypoxia (Vaux *et al*. 2001). Besides the mitochondrial respiratory chain, NADPH oxidase is another important source of ROS in hypoxic responses (Matsuzaki *et al*. 2005). Our results shown that the NADPH oxidase inhibitor DPI greatly reduced the HIF-1alpha accumulation under hypoxic condition (Fig. 1), suggesting an important role of NADPH oxidase in regulating HIF-1alpha.

It is possible that in addition to NADPH oxidase, other ROS generating enzymes, such as heme oxygenase-1 (Mancuso *et al*. 2006), cyclooxygenase isoforms and xanthine oxidase (Rieger *et al*. 2002) may also contribute toward HIF-1alpha regulation under hypoxic conditions. SOD mimetic MnTMPyP was used to remove total superoxide. Incubation of astrocytes with the MnTMPyP reduced hypoxia-induced HIF-1alpha protein accumulation, but to a much less extent than DPI, which inhibits the NADPH oxidase-mediated superoxide generation (Fig. 1). One plausible explanation is that H_2_O_2_, the product of the SOD catalytic reaction, is another important ROS species that contributes to the stabilization of HIF-1alpha (Marinho *et al*. 2014; Wang and MacNaughton 2005; Wang *et al*. 2006), thus offsetting some effect of superoxide removal.

Regulation of HIF-1alpha has also been considered an important mechanism for NO to modulate cellular responses to hypoxia. Previous studies addressing regulation of HIF-1 by NO has revealed a complex picture. Under normoxic conditions, NO was reported to stimulate HIF-1alpha accumulation. However, under hypoxic condition, NO was found to inhibit its DNA-binding activity and hypoxia-related gene expression (Agani *et al*. 2002; Kasuno *et al*. 2004; Sandau *et al*. 2001). In these studies, NO’s effects on the HIF-1alpha regulation were based on exogenous NO (NO donors). However, the role of endogenous NO production on HIF regulation has been largely neglected. Our results show that NO production is increased in astrocytes under hypoxic conditions (Fig. 2), which is consistent with previous studies (Fung *et al*. 2007; Hua *et al*. 2008). We found that scavenging NO with PTIO increased hypoxia-induced HIF-1alpha (Fig. 3). Moreover, removal of ROS by NAC dramatically decreased HIF-1alpha level, while the addition of PTIO to abolish NO effect reversed the NAC-induced reduction of hypoxia-induced HIF-1alpha (Fig. 5). These results demonstrate that endogenous NO inhibits HIF-1alpha expression under hypoxic conditions.

Interestingly, it was observed that NOS inhibitor L-NAME, in contrast to NO scavenger PTIO, decreased HIF-1alpha expression under hypoxic conditions (Fig. 3). This may be due to the fact that NOS is not only the source of NO, but also an important source of ROS. It is reported that under pro-oxidant conditions a reduction in tetrahydrobiopeterin due to oxidation results in the uncoupling of eNOS, leading to increased production of ROS, rather than NO (Landmesser *et al*. 2003). In addition to eNOS, nNOS was also reported to be a source of ROS in an environment lacking L-arginine (Weaver *et al*. 2005). In astrocyte cells, both eNOS and nNOS are present, and they could be a potential source of ROS (Hsiao *et al*. 2007; Marinho *et al*. 2014; Yuan *et al*. 2004). Analysis of intracellular ROS confirmed that L-NAME indeed decreased both superoxide (Fig. 4A, 4B and 4C) and H_2_O_2_ levels in the cells (Fig. 5D). It confirms that L-NAME not only blocked NOS, but also decreased superoxide. Thus, the reduction of HIF-1alpha level after L-NAME addition (Fig. 3) may be caused by the decreased ROS.

The rapid reaction between NO and superoxide produces peroxynitrite, which is another potent oxidant that causes cell injury and toxicity (Beckman *et al*. 1990). If peroxynitrite is the final effector responsible for the regulation of NO or ROS on HIF-1alpha expression, eliminating NO or ROS should show the same effects on HIF-1alpha accumulation, as both methods lead to decreased peroxynitrite production. Apparently, our results do not support this possibility because blocking NO and ROS showed opposite effects on HIF-1alpha expression. Furthermore, incubating astrocytes with authentic peroxynitrite did not change HIF-1alpha expression under hypoxic conditions (Fig. 6). These results suggest that NO and ROS, but not peroxynitrite, are the species responsible for the regulation of HIF-1alpha accumulation.

In conclusion, our results have demonstrated that, under hypoxic condition, endogenous ROS enhances the HIF-1alpha protein stabilization, while endogenous NO production weakens the stabilization. Since NO reacts with superoxide at near diffusion controlled rate (Nauser 2002), the interaction between NO and ROS may represent an important mechanism for controlling HIF-1alpha expression in response to hypoxic stress.

## MATERIALS AND METHODS

### Materials

L-NAME, PTIO, N-acetyl cysteine (NAC), Diphenylene iodonium (DPI), MnTMPyP, Dihydroethidium (DHE), 2',7'-dichlorodihydrofluorescein diacetate (DCFH-DA), 3-Morpholinosydnonimine hydrochloride (SIN-1) were purchased from Sigma (St. Louis, MO, USA). Dubbelco’s modified Eagles’s medium (DMEM) and Fetal bovine serum (FBS) were bought from Invitrogen (Carlsbad, CA, USA).

### Primary culture of rat cortical astrocytes

Rats were maintained and used in compliance with the principles set forth in the "Guide for Care and Use of Laboratory Animals" and approved by the University of New Mexico Animal Care and Use Committee. Primary cortical astrocytes were isolated from the cortices of postnatal day 1 Sprague–Dawley rat brains, as we described previously (Liu *et al*. 2007). The cells were harvested and placed through one round of enzymatic dissociation and expansion in astrocyte growth medium (DMEM containing 10% FBS, 100 units/mL penicillin, and 100 μg/mL streptomycin). When astrocytes reached subconfluence, cells were starved overnight in FBS-free DMEM before hypoxia treatment.

### C8-D1A astrocyte cell line culture

The C8-D1A astrocyte cell line obtained from American Type Culture Collection (ATCC, Manassas, VA, USA) were cultured in DMEM containing 10% FBS, penicillin (100 units/mL), and streptomycin (100 μg/mL). Medium was changed every three days, and the cells were starved overnight in FBS-free DMEM before experiments.

### Hypoxic cellular model

Before treatment, the cell culture medium was replaced FBS-free DMEM that was bubbled with 5% CO_2_/95% N_2_ for 30 min. Cells were then incubated in a humidified airtight chamber (Billups-Rothberg Inc., Del Mar, CA, USA) equipped with an air lock and flushed with 5% CO_2_/95% N_2_ for 15 min. The chamber was then sealed and kept at 37 °C for another 105 min. The oxygen concentration was below 0.2% as monitored by an oxygen analyzer (Sable Systems, Las Vegas, NV, USA).

### Measurement of NO concentration in the cultured medium

NO concentration in the conditioned medium was measured with the inNO-T nitric oxide measurement system (Warner Instruments, Hamden, CT, USA), using the amino-700 sensor as described previously (Gu *et al*. 2002). Briefly, the sensor was calibrated to the conversion of nitrite to NO in acidic solution in the presence of iodide ion. Then, after calibration, the sensor was inserted into the collected medium sample, the output current was monitored and the captured data was used to calculate the NO concentrations using the provided analysis software.

### Measuring intracellular ROS using fluorescent probes

Dihydroethidium (DHE) was used to measure intracellular superoxide levels, as DHE can enter the cells and be oxidized by superoxide to form ethidium, which produces fluorescence. After pre-incubation with DHE (5 μmol/L) for 20 min, cells were washed with PBS and then subjected to hypoxic treatment. At the end of hypoxic treatment, samples were collected, and fluorescence intensity was measured on a fluorescence plate reader (SpectraMax M_2_, Molecular Devices, Sunnyvale, CA, USA) at 510 nm excitation/590 nm emission to reflect intracellular superoxide production.

For the measurement of intracellular hydrogen peroxide levels, DCFH-DA was added to the cell culture medium (20 μmol/L) 20 min before and during hypoxic treatment. DFH-DA was converted to DCFH inside the cells and oxidized to DCF. Fluorescence intensity was measured at 485 nm excitation/ 535 nm emission to reflect intracellular hydrogen peroxide levels.

### Measuring intracellular superoxide using immuno-spin trapping

Immuno-spin trapping is a potent and sensitive method to detect protein-derived radicals produced. The reaction of superoxide with its spin trap 5,5-dimethylpyrroline-N-oxide(DMPO) is widely used to study superoxide production (Buettner 1993). Anti-DMPO antibody (Alexis Biochemicals, USA) was used to measure intracellular superoxide by immunofluorescence. 100 mmol/L DMPO was added just at the beginning of hypoxic treatment. At the end of hypoxic treatment, samples were collected, and then fixed in 4% PFA overnight at 4 °C. Cells were subsequently processed for immunostaining using rabbit anti-DMPO (1∶200), in order to reveal superoxide exposure. Alexa Fluor 488 Goat Anti-Rabbit IgG (Invitrogen, USA) was used to reveal the primary antibody. Fluorescence intensity was measured at 488 nm excitation/ 535 nm.

### Western-blot analysis of HIF-1alpha protein

At the end of the experiment, cells were quickly collected and lysed in RIPA buffer (Santa-Cruz). Cell extracts were homogenized by sonication and centrifuged at 14,000 *g* for 15 min at 4 °C. Protein concentrations were determined with protein assay reagent (Bio-Rad). Samples (50 μg of total protein) were boiled for 5 min and then electrophoresed in 8% SDS-PAGE acrylamide gels, transferred onto nitrocellulose membranes (Bio-Rad, Hercules, CA, USA), and incubated for 1 h in TBS-T (Tris-buffered saline and 0.1% Tween 20) containing 5% nonfat milk at room temperature. Membranes were then incubated overnight with rabbit polyclonal anti-HIF-1alpha antibody (1:1000 dilution; Novas Biologicals, Littleton, CO, USA), washed in TBS-T, incubated for 1 h at room temperature with HRP-conjugated anti-rabbit IgG (1:1000; Santa Cruz Biotech, Santa Cruz, CA, USA). The protein was detected using the SuperSignal West Pico chemiluminescent kit (Pierce, Rockford, IL, USA) according to the manufacturer’s instructions, and the bands were visualized and quantified on a Kodak Image Station 4000 Digital Imaging System (Carestream Molecular Imaging, New Haven, CT, USA). To control sample loading and protein transfer, the membranes were stripped and rehybridized for β-actin (1:4000, Santa Cruz Biotech).

### Statistical analysis

Results are expressed as mean ± SD. Statistical analysis was performed using ANOVA or Student’s *t*-test. A value of *p* < 0.05 was considered statistically significant.

## Abbreviations

HIF-1　　　 Hypoxia-inducible factor 1

NO　　　　 Nitric oxide

ROS　　　　 Reactive oxygen species

NOS　　　　 Nitric oxide synthase

DPI　　　　 Diphenylene iodonium

PHD　　　　Prolyl hydroxylase domain-containing 　　　　　　　 enzymes

NAC　　　　 N-acetyl cysteine

DCFH-DA　　 Dichlorodihydrofluorescein diacetate

DMEM　　　 Dubbelco's modified Eagles's medium

FBS　　　　 Fetal bovine serum

DHE　　　　 Dihydroethidium

## Conflict of interest

Qingquan Chen, Wenlan Liu, Xi Sun, Ke Jian Liu and Rong Pan declare that they have no conflict of interest.
